# Testicular organoids formation from leukaemia-infiltrated prepubertal testicular tissue: implications for fertility preservation

**DOI:** 10.1038/s41375-026-02938-x

**Published:** 2026-04-01

**Authors:** Yanhua Cui, Jouko Lohi, Cecilia Lindskog, Kirsi Jahnukainen, Jan-Bernd Stukenborg

**Affiliations:** 1 NORDFERTIL Research Lab Uppsala, Department of Organismal Biology, Uppsala University, Uppsala, Sweden; 2 Childhood Cancer Research Unit, Department of Women’s and Children’s Health, Karolinska Institutet, and Karolinska University Hospital, Solna, Sweden; 3 Department of Pathology, University of Helsinki and Helsinki University Hospital, Helsinki, Finland; 4 Department of Immunology, Genetics and Pathology, Cancer Precision Medicine Research Program, Uppsala University, Uppsala, Sweden; 5 New Children’s Hospital, Paediatric Research Centre, University of Helsinki and Helsinki University Hospital, Helsinki, Finland

**Keywords:** Paediatrics, Cell biology

## To the Editor:

Acute lymphoblastic leukaemia (ALL) in children and adolescents may affect the testes at diagnosis or relapse. Highly aggressive treatments such as haematopoietic stem cell transplantation are recommended for early testicular relapse and T-cell ALL. Patients with late isolated testicular relapse of B-cell ALL can generally be cured by salvage chemotherapy combined with scrotal irradiation or orchiectomy [1]. These treatments frequently lead to severe gonadal dysfunction and azoospermia, highlighting the need for fertility preservation. While postpubertal males can preserve fertility through semen cryopreservation, prepubertal boys do not yet produce sperm. As a result, fertility preservation for this group relies on cryopreservation of testicular tissue.

Currently, autotransplantation appears to be the most promising approach for the maturation of immature testicular tissue, as offspring have been obtained in non-human primates after testicular tissue transplantation [2]. However, when leukaemic cells infiltrate the testicular tissue, alternative fertility restoration methods are needed. In vitro spermatogenesis, including explant tissue and testicular organoid culture, shows promise but remains experimental. Further research is required before clinical application.

To optimise the cryobanking of prepubertal testicular tissue for patients with leukaemic testicular relapse, further research on the impact of leukaemic cells contamination on spermatogonia and the testicular somatic cell function is required. Reference values for spermatogonial quantity in human testes throughout healthy prepuberty have been established [3], and patient-derived testicular organoids and explant tissue cultures have proven valuable for studying somatic cell function [4, 5]. This study applied these advanced methods to assess the effects of overt and microscopic leukaemia contamination on the cellular composition and functionality of prepubertal testicular tissue.

**Fig. 1:**
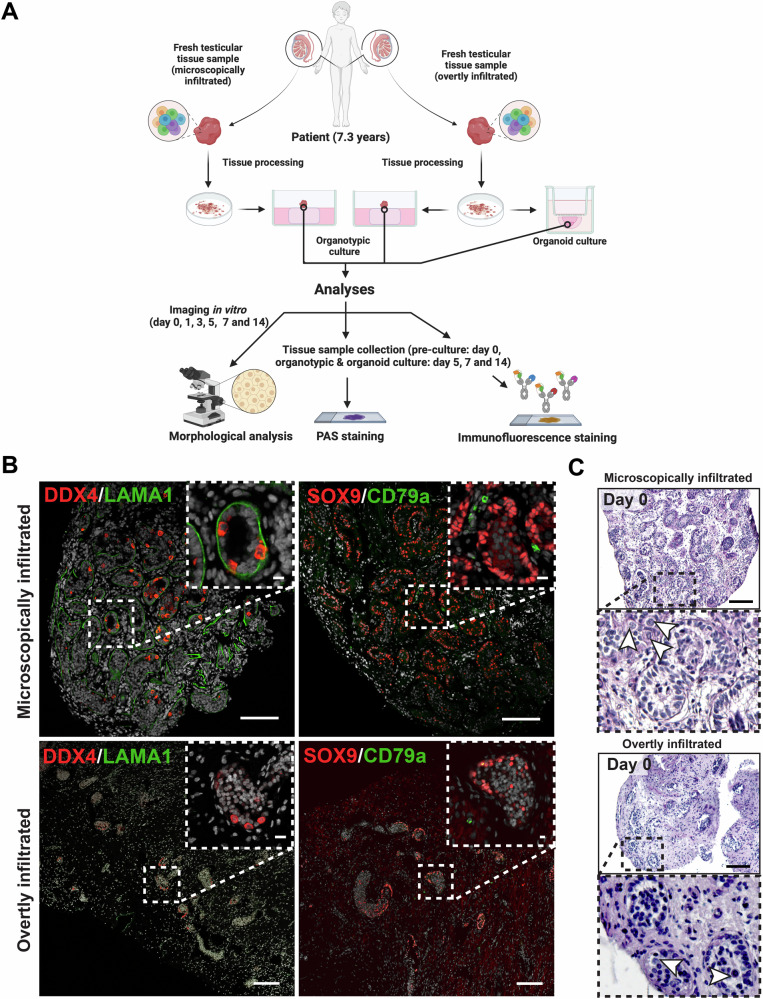
Characterisation of human prepubertal testicular tissue microscopically and overtly infiltrated with leukaemic cells. **A** Schematic illustration of the experimental conditions and characterisation analysis (created with BioRender®, Toronto, Canada). **B** Immunofluorescent staining of a tissue section depicting localisation of germ cell marker DDX4 (red staining), the basement membrane component LAMA1 (green staining), Sertoli cell marker SOX9 (red staining), and leukaemic cell marker CD79a (green staining). **C** Representative PAS staining images showing the histology of tissue samples from microscopically and overtly leukaemic cell infiltrated testes (white arrowheads: germ cells). Scale bars = 100 µm (insets = 20 µm). DDX4 DEAD-box helicase 4, LAMA1 laminin alpha 1, SOX9 SRY-Box transcription factor 9, WT1 Wilms’ Tumour 1, PAS Periodic Acid Schiff.

The study material comprised bilateral testicular samples from a prepubertal boy experiencing a combined bone marrow and testicular relapse of ALL. The patient had completed anti-leukaemia therapy one year before the relapse and received a three-week re-induction therapy course (Supplementary information) prior to a testicular biopsy for fertility preservation on normal-sized prepubertal testes measured volume: 1.8 ml, testicular volume Z-score: +0.5 SD, and an orchiectomy of an overtly enlarged testis (volume: 13.0 ml, Z-score: +4.4 SD) [6]. Two-thirds of the tissue obtained from the normal-sized testis was cryopreserved for clinical fertility preservation, while the remaining one-third, along with the entire orchiectomy specimen, was used in an experimental study under the NORDFERTIL fertility preservation programme (Fig. 1A, Supplementary Fig. 1). The patient and his parents received verbal and written information about the research project and provided written consent. Further details are provided in the Supplementary Information.

## Results

To identify leukaemic infiltration, testicular biopsy samples from both the enlarged and normal-sized testes were fixed in formalin, embedded in paraffin, and stained with immunohistochemical lymphoid cell markers, including TdT and CD79a, which were expressed on the patient’s leukaemic cells at the time of ALL diagnosis. Both the enlarged and normal-sized testes stained positively for the leukaemic cell markers TdT (Supplementary Fig. 2) and CD79a (Fig. 1C, Supplementary Fig. 2). Light microscopy examination of the overtly leukaemic testis showed mononuclear infiltration, fibrosis, and widened seminiferous tubules. In contrast, the contralateral testis displayed normal interstitial morphology without mononuclear infiltration (Fig. 1B). Furthermore, immunofluorescence analysis demonstrated the expression of DDX4 (germ cells) and SOX9 and WT1 (Sertoli cells) in both testicular tissue samples before and during culture (Fig. 1C; Supplementary Fig. 3). Notably, ECM protein marker LAMA1 expression was detected exclusively in the microscopically infiltrated testis tissue samples (Fig. 1C). The proportion of SOX9-positive to WT1-positive Sertoli cells (SOX9/WT1 ratio) was determined by counting the SOX9-positive and WT1-positive nuclei on each analysed cross-section. At biopsy, SOX9-positive cells accounted for 44.8% and 71.6% of the WT1-positive Sertoli cell population (SOX9/WT1) in the overtly and microscopically infiltrated testes, respectively, with no significant changes under organotypic or organoid culture conditions (Supplementary Fig. 4).

Spermatogonia were identified based on morphology, localization within seminiferous cords, and DDX4 positivity [7]. At least 25 round seminiferous cords were analysed to determine spermatogonia per tubule/cord cross-section (S/T) and the fertility index (FI). According to published reference data, S/T Z-scores within ±3 SD indicate normal spermatogonial numbers, whereas Z-scores below −7 SD indicate marked spermatogonial depletion [8]. The S/T values in both the overtly and microscopically infiltrated leukaemic testes were comparable (1.46 and 0.89, respectively) and remained within the normative reference range with corresponding S/T Z-scores of −1.3 and −3.0, respectively. Similarly, the fertility index (FI) was comparable between the overtly and microscopically infiltrated leukaemic testes (46.15% vs. 46.43%, respectively).

**Fig. 2:**
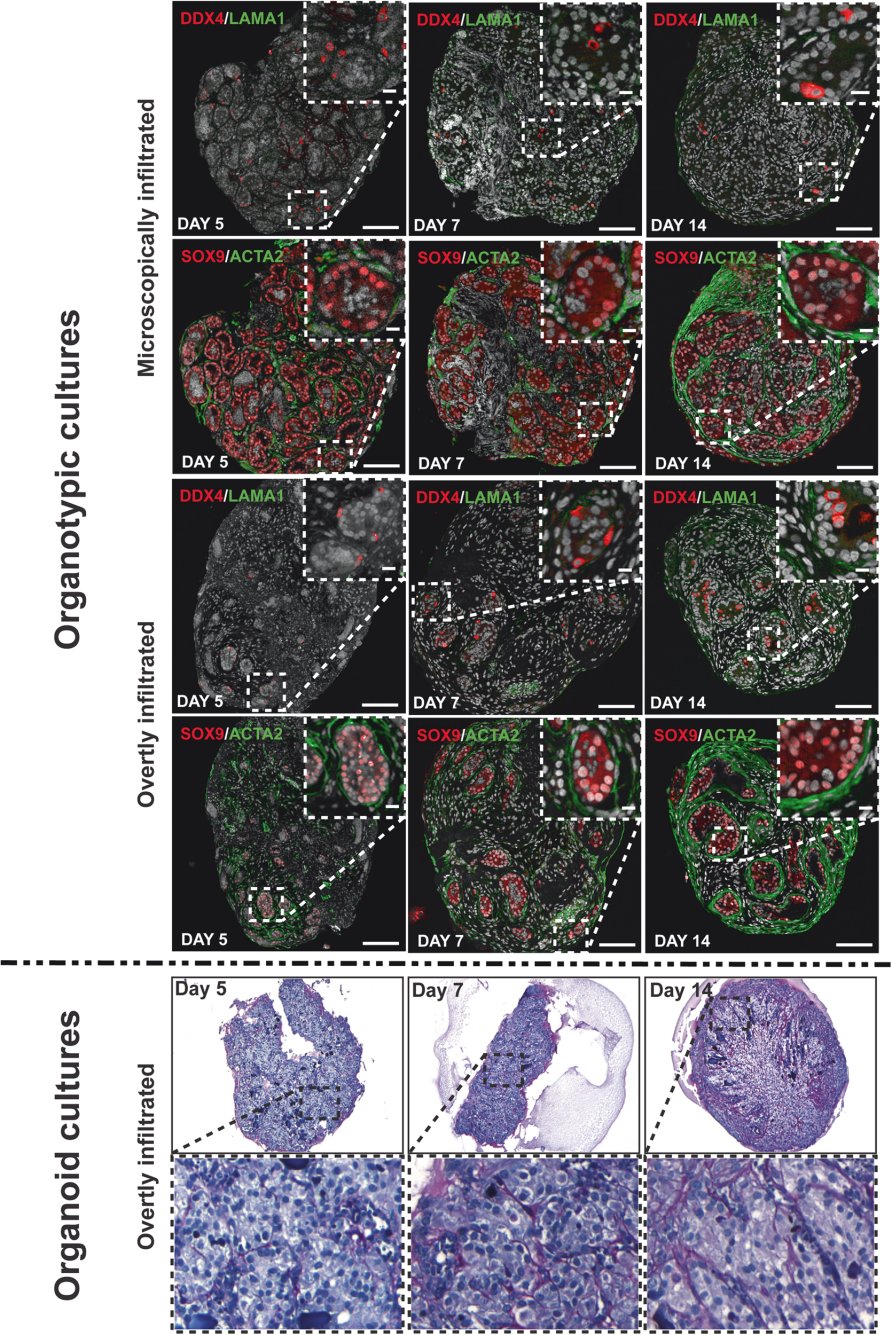
Characterization of human prepubertal testicular tissue microscopically and overtly infiltrated with leukaemic cells following culture for five, seven, and 14 days. Immunofluorescent staining of testicular tissue fragments cultured for up to 14 days in organotypic culture conditions, depicting localisation of Sertoli cell marker SOX9 (red staining), the peritubular cell marker ACTA2 (green staining), localisation of germ cell marker DDX4 (red staining) and the basement membrane component LAMA1 (green staining); Scale bars = 100 µm (insets = 20 µm). Representative PAS staining images showing the histology of testicular organoids generated from overtly leukaemic cell infiltrated testes. SRY-Box transcription factor 9 (SOX9), DEAD-box helicase 4 (DDX4), actin alpha 2 (ACTA2), laminin alpha 1 (LAMA1), Periodic Acid Schiff (PAS).

Fresh tissue samples from both enlarged and normal-sized testes were cultured for 14 days using an established testicular organotypic culture method (Figs. 1A, 2) [4]. Additionally, fresh tissue samples from the overtly enlarged testis were cut into small tissue fragments (approximately 1 mm³), enzymatically digested into a cell suspension, and applied to a three-layer gradient culture system (3-LGS) for organoid culture [5]. Following 14 days of organotypic culture, both microscopically and overtly infiltrated testis samples express DDX4 (germ cells), SOX9 (Sertoli cells), and ACTA2 (peritubular cells) at day 5, day 7 and day 14 (Supplementary Fig. 3). LAMA1 expression was detected in some samples of the overtly infiltrated testes at day 7 and day 14 (Fig. 2). After 14 days culture, primary cells isolated from the enlarged leukaemic testis generated testicular organoids, as confirmed by histological analysis (Supplementary Fig. 1). These organoids exhibited compartmentalised seminiferous cord-like and interstitial-like structures, closely replicating the tissue structure observed in explant-tissue culture at the same time point. The spatial distribution and cellular composition of the testicular organoids (Fig. 2) were consistent with those previously reported in organoids derived from paediatric cancer patients [5]. SOX9 and WT1 signals were clearly observed in testicular organoids, confirming successful Sertoli cell integration (Supplementary Fig. 4). In contrast, signals for the structural markers ACTA2, or LAMA1 were not detectable. Leydig cells in both primary cell suspensions and explant cultures from the overtly enlarged leukaemic testis produced testosterone, indicating preserved Leydig cell function (Supplementary Fig. 5). No morphological evidence of leukaemic infiltration was observed in any of the explant tissues or testicular organoid cultures (Fig. 2). Additionally, lymphoid marker CD79a remained negative after the first five days of organotypic culture (Supplementary Fig. 6).

## Discussion

Current fertility preservation strategies for prepubertal patients focus on the cryopreservation of testicular tissue samples containing spermatogonial stem cells [9]. Anti-leukaemia therapy involving alkylating agents can significantly reduce the spermatogonia number in testicular tissue harvested for fertility preservation. Cumulative cyclophosphamide equivalent (CED) doses exceeding 4 g/m² have been reported to deplete S/T values [7, 10]. In the present analysis, S/T Z-scores remained within the normative reference range ( ± 3 SD), following exposure to a CED dose of 1 g/m², supporting a limited impact of low-dose alkylating agent exposure on spermatogonial numbers.

Recently, reduced spermatogonial numbers have been reported in pre- and peripubertal cancer patients prior to gonadotoxic therapy when compared to a simulated control cohort [11, 12]. However, a sub-analysis using immunohistochemistry and mutation-specific PCR showed no significant differences in S/T Z-scores between leukaemic cell-infiltrated and non-infiltrated testicular tissue. The current finding that both overtly and microscopically infiltrated leukaemic testes post-induction chemotherapy retain spermatogonia within age-adjusted reference ranges supports the conclusion that leukaemic cell contamination does not significantly compromise the spermatogonial pool.

In the present study, the overtly leukaemic testis successfully formed organoids capable of producing testosterone, indicating preserved Sertoli and Leydig cell function. This contrasts with previous reports in which over 50% of testicular cell samples from prepubertal cancer patients failed to generate organoids, pointing to deficiencies in the testicular somatic cell functioning [5]. SOX9 expression was observed in 44.8% of the WT1-positive Sertoli cells in the leukaemic testis, supporting the previously proposed threshold of >49.4% SOX9-positive Sertoli cells as a predictor of organoid-forming potential [5].

Currently, no reliable method exists to eliminate malignant cells from testicular tissue. In this study, induction chemotherapy markedly reduced the leukaemic burden, with only rare residual cells observed in the interstitium, corresponding to a minimal residual disease level of 0.2% in the bone marrow. These findings suggest that testicular tissue collection after induction therapy may lower the risk of contamination. Furthermore, no residual leukaemic cells were detected after the first five days of tissue culture. However, the histological and immunohistochemical methods used in the current study lack the sensitivity necessary for accurate risk assessment, while more sensitive approaches, such as mutation-specific PCR, would have required larger, well-preserved tissue samples. It can be speculated that the loss of CD79a-positive leukaemic cells in organotypic cultures is driven by an unsupportive somatic microenvironment. The xeno-free medium in our experiments lacks niche-specific survival signals and may promote cellular stress and apoptosis. Collectively, these conditions may create an adverse environment that contributes to the elimination of leukaemic cells. Further research is required to elucidate these findings in detail.

In summary, this is the first report of successful organoid formation from overtly leukaemic prepubertal testicular tissue, demonstrating a normal spermatogonial pool and functional Sertoli and Leydig cells. The results indicate that overt leukaemic infiltration does not impair the functional capacity of the testicular somatic environment or diminish the tissue’s potential for fertility restoration. Consequently, if orchidectomy is performed to consolidate therapy for testicular relapse, the collected testicular material could be cryopreserved for fertility preservation purposes. Proper counselling should be provided regarding the presence of leukaemic contamination, including information that current protocols are insufficient to eliminate cancer cells from harvested gonadal tissue. Further research is needed to develop alternative clinical strategies for testicular tissue autotransplantation to restore fertility in male leukaemia patients.

## Supplementary information

Supplementary information - figure_table legends

Supplementary Table S1 - Primary and secondary antibodies

Supplementary Figure S1 - Generation of human prepubertal testicular organoids (TOs) with cells obtained from an overtly infiltrated testis with leukaemic cells.

Supplementary Figure S2 - Characterisation of human prepubertal testicular tissue microscopically and overtly infiltrated with leukaemic cells.

Supplementary Figure S3 - Quantitative analysis of germ and Sertoli cell populations in organotypic cultures from microscopically or overtly infiltrated testicular tissue.

Supplementary Figure S4 - Expression of WT1 and SOX9 in testicular organotypic and organoid cultures.

Supplementary Figure S5 - Testosterone levels in organotypic and organoid cultures.

Supplementary Figure S6 - Immunofluorescence analysis of leukaemic cell persistence in organotypic cultures.

## References

[1] Nguyen HTK, Terao MA, Green DM, Pui CH, Inaba H. Testicular involvement of acute lymphoblastic leukemia in children and adolescents: diagnosis, biology, and management. Cancer. 2021;127:3067–81. 34031876 10.1002/cncr.33609 PMC9677247

[2] Fayomi AP, Peters K, Sukhwani M, Valli-Pulaski H, Shetty G, Meistrich ML, et al. Autologous grafting of cryopreserved prepubertal rhesus testis produces sperm and offspring. Science. 2019;363:1314–9. 30898927 10.1126/science.aav2914 PMC6598202

[3] Masliukaite I, Hagen JM, Jahnukainen K, Stukenborg JB, Repping S, van der Veen F, et al. Establishing reference values for age-related spermatogonial quantity in prepubertal human testes: a systematic review and meta-analysis. Fertil Steril. 2016;106:1652–7.e1652. 27717555 10.1016/j.fertnstert.2016.09.002

[4] Kurek M, Akesson E, Yoshihara M, Oliver E, Cui Y, Becker M, et al. Spermatogonia loss correlates with LAMA 1 expression in human prepubertal testes stored for fertility preservation. Cells. 2021;10:241. 33513766 10.3390/cells10020241 PMC7911157

[5] Cui Y, Harteveld F, Ba Omar HAM, Yang Y, Bjarnason R, Romerius P, et al. Prior exposure to alkylating agents negatively impacts testicular organoid formation in cells obtained from childhood cancer patients. Hum Reprod open. 2024;2024:hoae049. 39188568 10.1093/hropen/hoae049 PMC11346771

[6] Koskela M, Virtanen HE, Rodprasert W, Jahnukainen K, Toppari J, Koskenniemi JJ. Pubertal testicular volume references for ruler, orchidometer, and ultrasonography measurements based on a longitudinal follow-up. Andrology. 2024;12:1771–9. 38482926 10.1111/andr.13629

[7] Stukenborg JB, Alves-Lopes JP, Kurek M, Albalushi H, Reda A, Keros V, et al. Spermatogonial quantity in human prepubertal testicular tissue collected for fertility preservation prior to potentially sterilizing therapy. Hum Reprod. 2018;33:1677–83. 30052981 10.1093/humrep/dey240 PMC6112575

[8] Funke M, Yang Y, Lahtinen A, Benninghoven-Frey K, Kliesch S, Neuhaus N, et al. Z-scores for comparative analyses of spermatogonial numbers throughout human development. Fertil Steril. 2021;116:713–20. 33975728 10.1016/j.fertnstert.2021.04.019

[9] Duffin K, Neuhaus N, Andersen CY, Barraud-Lange V, Braye A, Eguizabal C, et al. A 20-year overview of fertility preservation in boys: new insights gained through a comprehensive international survey. Hum Reprod Open. 2024;2024:hoae010. 38449521 10.1093/hropen/hoae010 PMC10914450

[10] Poganitsch-Korhonen M, Masliukaite I, Nurmio M, Lahteenmaki P, van Wely M, van Pelt AMM, et al. Decreased spermatogonial quantity in prepubertal boys with leukaemia treated with alkylating agents. Leukemia. 2017;31:1460–3. 28270690 10.1038/leu.2017.76 PMC5467043

[11] Masliukaite I, Ntemou E, Feijen EAM, van de Wetering M, Meissner A, Soufan AT, et al. Childhood cancer and hematological disorders negatively affect spermatogonial quantity at diagnosis: a retrospective study of a male fertility preservation cohort. Hum Reprod. 2023;38:359–70. 36708005 10.1093/humrep/dead004 PMC9977127

[12] Kourta D, Camboni A, Saussoy P, Kanbar M, Poels J, Wyns C. Evaluating testicular tissue for future autotransplantation: focus on cancer cell contamination and presence of spermatogonia in tissue cryobanked for boys diagnosed with a hematological malignancy. Hum Reprod. 2024;39:486–95. 38227814 10.1093/humrep/dead271

