# Supplementary material for: Testicular organoids formation from leukaemia-infiltrated prepubertal testicular tissue: implications for fertility preservation
Source: Leukemia. 2026 Apr 1;40(5):1044–8. doi: 10.1038/s41375-026-02938-x (PMC13148997; doi:10.1038/s41375-026-02938-x)
Supplement: Supplementary file 1 — Supplementary information - figure_table legends [file 41375_2026_2938_MOESM1_ESM.docx]

**Supplementary information**

# **Title:** Testicular Organoids Formation from Leukaemia-Infiltrated Prepubertal Testicular Tissue: Implications for Fertility Preservation

# Running title: **Organoids from leukaemic prepubertal testis**

# **Authors:** Yanhua Cui^1,2^, Jouko Lohi^3^, Cecilia Lindskog^4^, Kirsi Jahnukainen^1,2,5,*,#^, Jan-Bernd Stukenborg^1,2,*,#^

**Affiliations:** ^1^NORDFERTIL Research Lab Uppsala, Department of Organismal Biology, Uppsala University, Uppsala, Sweden, ^2^Childhood Cancer Research Unit, Department of Women's and Children's Health, Karolinska Institutet, and Karolinska University Hospital, Solna, Sweden; ^3^Department of Pathology, University of Helsinki and Helsinki University Hospital, Finland, ^4^Department of Immunology, Genetics and Pathology, Cancer Precision Medicine Research Program, Uppsala University, Uppsala, Sweden; ^5^New Children's Hospital, Paediatric Research Centre, University of Helsinki and Helsinki University Hospital, Helsinki, Finland,

*Correspondence: Kirsi Jahnukainen, MD, PhD, Division of Haematology-Oncology and Stem Cell Transplantation, Children's Hospital, University of Helsinki, FIN-00029 HUS, Finland. E-mail: [kirsi.jahnukainen@hus.fi](mailto:kirsi.jahnukainen@hus.fi), Tel: +358-9-4711 or Jan-Bernd Stukenborg, PhD, NORDFERTIL Research Lab Uppsala, Department of Organismal Biology, Uppsala University, Uppsala, Sweden, Norbyvägen 18A, 752 36 Uppsala, Sweden; email: [jan-bernd.stukenborg@ebc.uu.se](mailto:jan-bernd.stukenborg@ebc.uu.se)

^#^Both authors contributed equally to the work presented.

**Materials and methods**

**Ethical approval**

Ethical approval for the use of testicular tissues was obtained from the Ethics Board of the University of Helsinki (426/13/03/03/2015) and the Regional Ethics Board in Stockholm (Dnr 2013-2129-31-3, and Dnr 2021-04277).

**Previous exposure to cancer therapy and medical status at testicular biopsy**

The patient had concluded anti-leukaemia therapy one year before the relapse, which included the NOPHO ALL 2008 IR protocol with a cumulative cyclophosphamide equivalent dose of 1000 mg/m² and a cumulative doxorubicin dose of 80 mg/m² ^1, 2^. Following the testicular relapse and prior to the testicular biopsy, the patient received a three-week re-induction therapy course (SIB) from the IntReALL SR 2010 protocol, including dexamethasone 200 mg/m², vincristine 4.5 mg/m², mitoxantrone 20 mg/m², PEG-asparaginase 1000 IU/m², and two doses of intrathecal methotrexate. No significant bleeding or infection risks, which were exclusion criteria for the study, were identified. Bone marrow analysis revealed minimal residual leukaemia at 0.2% as determined by flow cytometry.

**Testicular organotypic cultures**

Prepubertal testicular tissue samples were cultured by an established testicular explant culture method ^3^. Testicular biopsy samples were cut into approximately 1mm^3^ small tissue fragments, then placed on top of a 0.35% agarose block in a 6-well plate to create an air-liquid interface explant tissue culture system. Of these, three tissue fragments for each block, and 3 blocks for each well. The agar blocks were prepared by mixing the 0.7% SeaKem® LE agarose (50004, Lonza, Basel, Switzerland) and NutriStem (05-100-1, Biological Industries, Kibbutz Beit-Haemek, Israel) with 1% penicillin and streptomycin (15070-063, Gibco, Grand Island, NY, USA) in a ratio of 1:1. To make the agar block, 1mL of the microwave heated mixture was pipetted into the 24-well plates. After 15 minutes, the blocks were solidified and removed from the 24-well plates by the proximal end of the 1mL pipette tips (0832121, Biosphere Plus, Thermo Fisher Scientific, Waltham, MA, USA). The testicular tissue pieces were cultured in NutriStem (05-100-1, Biological Industries) with 10% KSR (KnockOut serum replacement XenoFree; 10828-028, Gibco), 1% penicillin and streptomycin (Gibco, 15070-063) and 10-7 M melatonin (M5250-1G, Sigma-Aldrich, St. Louis, MO, USA) in a 6-well at 35 ℃ for 14 days with a media change every week. Tissue and media samples were collected on day 1, 5, 7 and day 14 of the culture.

**Three-layer gradient system (3LGS)**

Small tissue fragments (approximately 1 mm^3^) of testicular tissue samples were enzymatically digested into a cell suspension and then applied to the organoid culture system. The 3-LGS consists of a layer of testicular cells suspended in Matrigel, placed between two cell-free Matrigel layers. Testicular organoids were cultured in NutriStem (05-100-1, Biological Industries) with 10% KSR (KnockOut serum replacement XenoFree; 10828-028, Gibco), 1% penicillin and streptomycin (15070-063, Gibco) and 10^-7^ M melatonin (M5250-1G, Sigma-Aldrich) in a 24-well plate (734-2325, VWR,  Stockholm, Sweden), for 7 days at 35 ̊C with a media change every 48 hours.

**Periodic Acid Schiff (PAS) staining**

Testicular organoids and tissue fragments were fixed in 4% PFA (HL96753.1000, Histolab, Askim, Sweden) or Bouin's solution (HT10132, Sigma-Aldrich). After dehydration, samples were embedded in paraffin (Paraplast, P3808, Sigma-Aldrich) and cut into 5-µm thick sections using a microtome (HM350, Microm, Heidelberg, Germany). For morphology analysis, paraffin-embedded organoids and tissue fragments were stained with a Periodic Acid-Schiff staining kit (101646, Sigma-Aldrich). Briefly, sections were deparaffinised in xylene (1330-20-7, Fisher Scientific, Hampton, NH, USA) and rehydrated in a descending ethanol series: 100% ethanol (01399.01L, Histolab), 96% ethanol (01396.01L, HistoLab), and 70% ethanol (01370.01L, HistoLab). Then sections were oxidised in 0.5% Periodic Acid solution for 5 minutes, incubated in Schiff's reagent for 15 minutes, and counterstained in Hematoxylin solution (105175, Sigma-Aldrich) for one minute. Between each step, the sections were rinsed in distilled water. Finally, the sections were dehydrated and mounted using the xylene-based mounting agent Pertex (1721183, Histolab). Images were obtained with a bright field microscope (DFC290, Leica, Wetzlar, Germany).

**Immunofluorescence staining**

For immunofluorescence analysis, three sections per slide were subjected to heat-mediated antigen retrieval at 95 ºC in 10 mM sodium citrate (C7254, Sigma-Aldrich) with a pH of 6. Subsequently, samples were blocked in tris-buffered saline (TBS) supplemented with 10% normal donkey serum (017-000-121, Jackson Immuno Research, West Grove, PA, USA) and 1% bovine serum albumin (A2153, Sigma-Aldrich). Primary antibodies (Supplementary Tab. S1) were diluted in the blocking buffer and slides were incubated overnight at 4 ºC. Negative controls were performed with mouse and rabbit IgGs (Supplementary Tab. S1). After three washing steps in TBS for five minutes each, samples were incubated at room temperature with fluorescence-conjugated secondary antibodies (Supplementary Tab. S1) and DAPI (135-1303, Bio-Rad, Hercules, CA, USA) diluted in the blocking buffer., before mounting with an anti-fade mounting medium (P36931, Invitrogen, Waltham, MA, USA). Immunofluorescence images were taken with a confocal microscope (LSM700, Zeiss, Jena, German).

**Immunohistochemistry (IHC) and hematoxylin–eosin (HE) staining**

To assess leukaemic infiltration, testicular biopsy specimens from both the enlarged and contralateral normal-sized testis were formalin-fixed, paraffin-embedded, and processed for HE staining and IHC with lymphoid markers TdT (SEN28, Novocastra, Newcastle, UK) and CD79a (SP18, Roche/Ventana, Tucson, AZ, USA), previously verified on the patient’s leukemic blasts at initial ALL diagnosis. IHC was performed at the Department of Pathology, Helsinki University Hospital, following standard protocols, using the UltraView Universal DAB Detection Kit (760-500, Roche/Ventana) for CD79a and the OptiView DAB IHC Detection Kit (760-700, Roche/Ventana) for TdT.

**Testosterone assay**

To evaluate hormone production, media samples were analyzed by specific enzyme-linked immunosorbent assays (ELISAs) for testosterone (EIA-1559, AH diagnostics, Solna, Sweden) according to the manufacturer’s instructions. The FLUO Star Omega microplate reader (BMG LABTECH, Offenburg, Germany) and MARS Data Analysis software, version 2.10 R3 (BMG LABTECH) were used to measure absorbance. Testosterone concentrations were calculated using a Four-Parameter Logistic curve fit.

**Quantification and Statistics**

Quantification and statistical analysis of the immunofluorescence images was performed using QuPath (v.0.5.0). The analytical workflow was executed as follows: Cell Segmentation: Automated detection of all DAPI-positive cells on each tissue section was achieved using the InstanceSeg extension. Segmentation was guided by the fluorescence_nuclear_and_cells model. Object Classification: A Single Measurement Classifier was selected to categorize the segmented cells based on their specific immunofluorescence (DDX4, SOX9, WT1, and CD79a). By defining threshold for each fluorescence channel, cells were systematically classified as either positive or negative. Data are presented as mean ± SD. Statistical analysis using GraphPad Prism software (GraphPad Prism 7, Boston, MA, USA) was performed using one-way ANOVA followed by Kruskal-Wallis test and multiple comparisons test comparing each time point to day 0. P < 0.05 was considered statistically significant.

**Supplementary Table S1: Primary and secondary antibodies**. Abbreviations: SRY-Box transcription factor 9 (SOX9), DEAD-box helicase 4 (DDX4), actin alpha 2 (ACTA2), laminin alpha 1 (LAMA1), Terminal Desoxynucleotidyl Transferase (TdT), Cluster of Differentiation 79 alpha (CD79a), Wilms' Tumor 1 (WT1), Immunoglobolin G (IgG), Cyanine 3 (Cy3).

**Supplementary Figure S1: Generation of human prepubertal testicular organoids (TOs) with cells obtained from an overtly infiltrated testis with leukaemic cells.** Schematic illustration of the study design (created with BioRender®). Representative images of the human prepubertal testicular cells reorganisation into single aggregates. Scale bars = 500µm.

**Supplementary Figure S2: Characterisation of human prepubertal testicular tissue microscopically and overtly infiltrated with leukaemic cells.** Representative HE staining images showing the histology of tissue samples from microscopically and overtly leukaemic cell infiltrated testes. Leukaemic cells are identified with TdT and CD79a antibodies (expression shown as brown staining). Scale bars = 100µm (insets = 10µm). Abbreviations: Terminal Desoxynucleotidyl Transferase (TdT), Haematoxylin-Eosin (HE), Cluster of Differentiation 79 alpha (CD79a).

**Supplementary Figure S3:** **Quantitative analysis of germ and Sertoli cell populations in organotypic cultures from microscopically or overtly infiltrated testicular tissue.** DDX4+ (germ cells), SOX9+ (Sertoli cells), and WT1+ (Sertoli cells) populations across the 14-day culture period (Day 0, 5, 7, and 14). Data are presented as the ratio of positive cell counts to the total number of cells identified per tissue section. Statistical significance among timepoints was determined using the Kruskal–Wallis test (*P < 0.05). n =3. Abbreviations: SRY-Box transcription factor 9 (SOX9), DEAD-box helicase 4 (DDX4), and Wilms' Tumor 1 (WT1).

**Supplementary Figure S4: Expression of WT1 and SOX9 in testicular organotypic and organoid cultures. A.** Representative double staining image of the organotypic and organoid cultures from overtly infiltrated testicular tissue at Days 0, 5, 7, and 14, Sertoli cells are labelled with WT1 (red staining) and SOX9 (green staining), Scale bars: 100µm (insets: 20µm). **B.** The percentage proportion of SOX9-positive cells within the WT1-positive Sertoli cell population (SOX9/WT1) across culture timepoints are shown for organotypic cultures from overtly and microscopically infiltrated testicular tissue and for organoid cultures from overtly infiltrated tissue. Abbreviations: SRY-Box transcription factor 9 (SOX9), Wilms' Tumor 1 (WT1), 4′,6-diamidino-2-phenylindole (DAPI).

**Supplementary Figure S5: Testosterone levels in organotypic and organoid cultures.** Testosterone concentrations (ng/ml) were measured in culture media of the organotypic and organoid cultures derived from overtly infiltrated testicular tissue at Days 0, 5, 7, and 14. Statistical significance across timepoints was assessed using the Kruskal–Wallis test. **P < 0.01, *P < 0.05. Abbreviations: Non-cultured media (NC-M)

**Supplementary Figure S6:** **Immunofluorescence analysis of leukaemic cell persistence in organotypic cultures.** Sertoli cell marker WT1 (red staining) and the leukaemic cell marker CD79a (yellow staining) expression was assessed at Days 0, 5, 7, and 14 (representative images shown for Day 0). Scale bars: 100µm (insets = 20µm). Quantification shows the percentage proportion of CD79a-positive cells relative to total DAPI-positive nuclei across culture timepoints in explant cultures from overtly and microscopically infiltrated testicular tissue. Abbreviations: Wilms' Tumor 1 (WT1), 4′,6-diamidino-2-phenylindole (DAPI), Cluster of Differentiation 79 alpha (CD79a).

**Reference:**

1. Green DM, Nolan VG, Goodman PJ, Whitton JA, Srivastava D, Leisenring WM*, et al.* The cyclophosphamide equivalent dose as an approach for quantifying alkylating agent exposure: a report from the Childhood Cancer Survivor Study. *Pediatr Blood Cancer* 2014 Jan; **61**(1)**:** 53-67.

2. Feijen EAM, Leisenring WM, Stratton KL, Ness KK, van der Pal HJH, van Dalen EC*, et al.* Derivation of Anthracycline and Anthraquinone Equivalence Ratios to Doxorubicin for Late-Onset Cardiotoxicity. *JAMA Oncol* 2019 Jun 1; **5**(6)**:** 864-871.

3. Sato T, Katagiri K, Gohbara A, Inoue K, Ogonuki N, Ogura A*, et al.* In vitro production of functional sperm in cultured neonatal mouse testes. *Nature* 2011; **471**(7339)**:** 504-507.
