# Supplementary material for: Testicular organoids formation from leukaemia-infiltrated prepubertal testicular tissue: implications for fertility preservation
Source: Leukemia. 2026 Apr 1;40(5):1044–8. doi: 10.1038/s41375-026-02938-x (PMC13148997; doi:10.1038/s41375-026-02938-x)
Supplement: Supplementary file 2 — Supplementary Table S1 - Primary and secondary antibodies [file 41375_2026_2938_MOESM2_ESM.docx]

**Supplementary Table S1: Primary and secondary antibodies**

| **Protein** | **Host**  **Species** | **Antibody concentration** | **Antibody dilution** | **Catalogue number** | **Brand** |
| --- | --- | --- | --- | --- | --- |
| **SOX9** | mouse | 0.5 mg/mL | 1:400 | ab76997 | Abcam, Cambridge, UK |
| **SOX9** | rabbit | 1 mg/mL | 1:400 | ab5535 | Sigma-Aldrich |
| **WT1** | rabbit | 0.221 mg/mL | 1:200 | ab89901 | Abcam |
| **DDX4** | rabbit | 1 mg/mL | 1:200 | ab27591 | Abcam |
| **ACTA2** | mouse | 6.0 mg/mL | 1:500 | a2547 | Sigma-Aldrich |
| **LAMA1** | mouse | 0.7 mg/mL | 1:100 | ab210954 | Abcam |
| **TdT** | rabbit | 0.2 mg/mL | 1:100 | 6051079 | Novocastra |
| **CD79a** | rabbit | 0.2 mg/mL | 1:100 | 05640296001 | Roche/Ventana |
| **Mouse IgG** | mouse | 0.4 mg/ml | 1:100 | sc-2025 | Santa Cruz Biotechnology, Dallas, TX, USA |
| **Rabbit IgG** | rabbit | 1.775 mg/ml | 1:200 | ab172730 | Abcam |
| **Goat IgG** | goat | 1 mg/ml | 1:200 | AB-108-C | R&D Systems, Minneapolis, MN, USA |
| **Cy3** | rabbit | 1.5 mg/ml | 1:500 | 711-166-152 | Jackson ImmunoResearch |
| **Alexa Fluor 488** | mouse | 1.5 mg/ml | 1:500 | 715-546-150 | Jackson ImmunoResearch |

Abbreviations: SRY-Box transcription factor 9 (SOX9), DEAD-box helicase 4 (DDX4), actin alpha 2 (ACTA2), laminin alpha 1 (LAMA1), Terminal Desoxynucleotidyl Transferase (TdT), Cluster of Differentiation 79 alpha (CD79a), Wilms' Tumor 1 (WT1), Immunoglobolin G (IgG), Cyanine 3 (Cy3).
