# Supplementary figures and images for: Testicular organoids formation from leukaemia-infiltrated prepubertal testicular tissue: implications for fertility preservation
Source: Leukemia. 2026 Apr 1;40(5):1044–8. doi: 10.1038/s41375-026-02938-x (PMC13148997; doi:10.1038/s41375-026-02938-x)

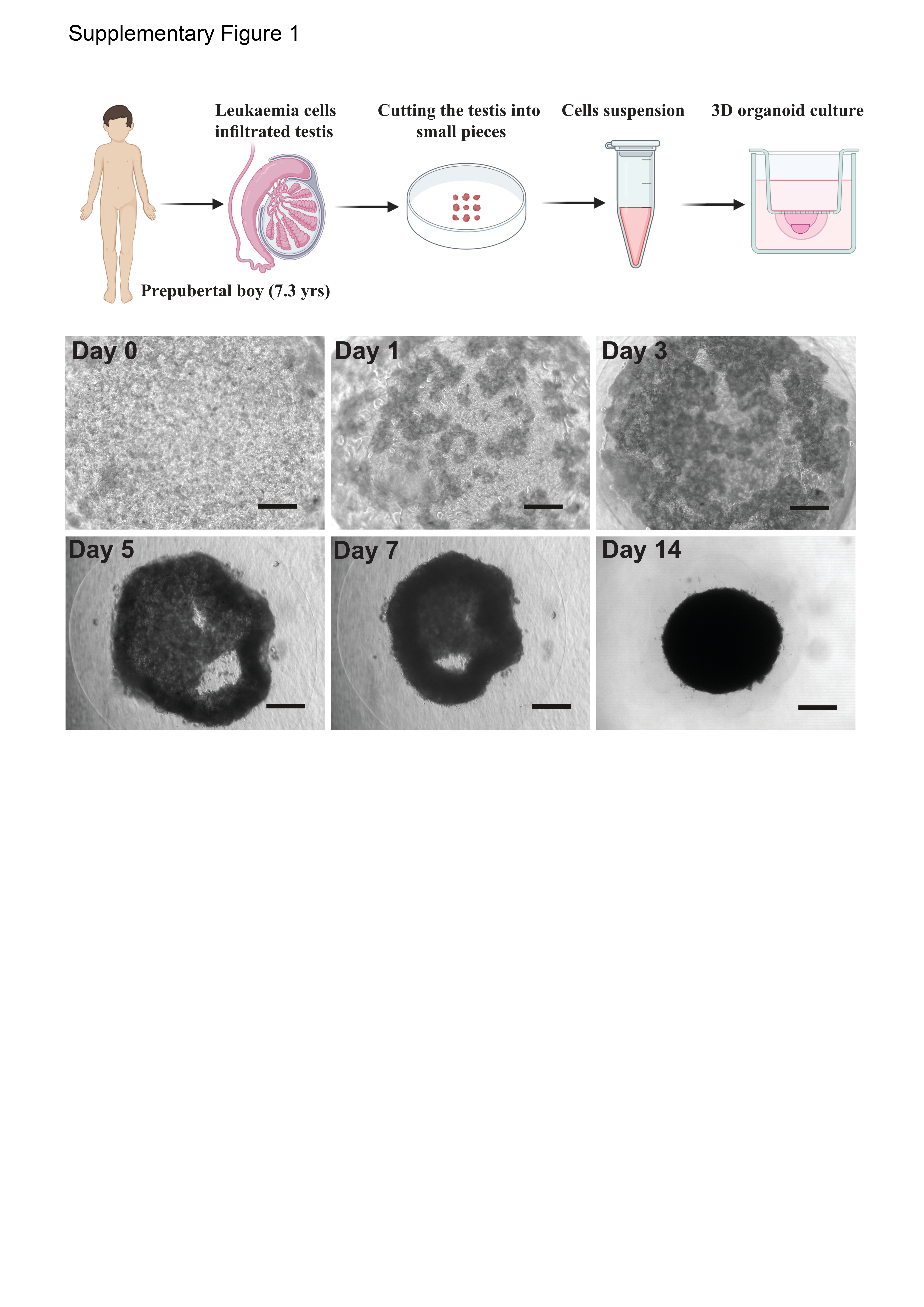

Supplement: Supplementary file 3 — Supplementary Figure S1 - Generation of human prepubertal testicular organoids (TOs) with cells obtained from an overtly infiltrated testis with leukaemic cells. [file 41375_2026_2938_MOESM3_ESM.tif]

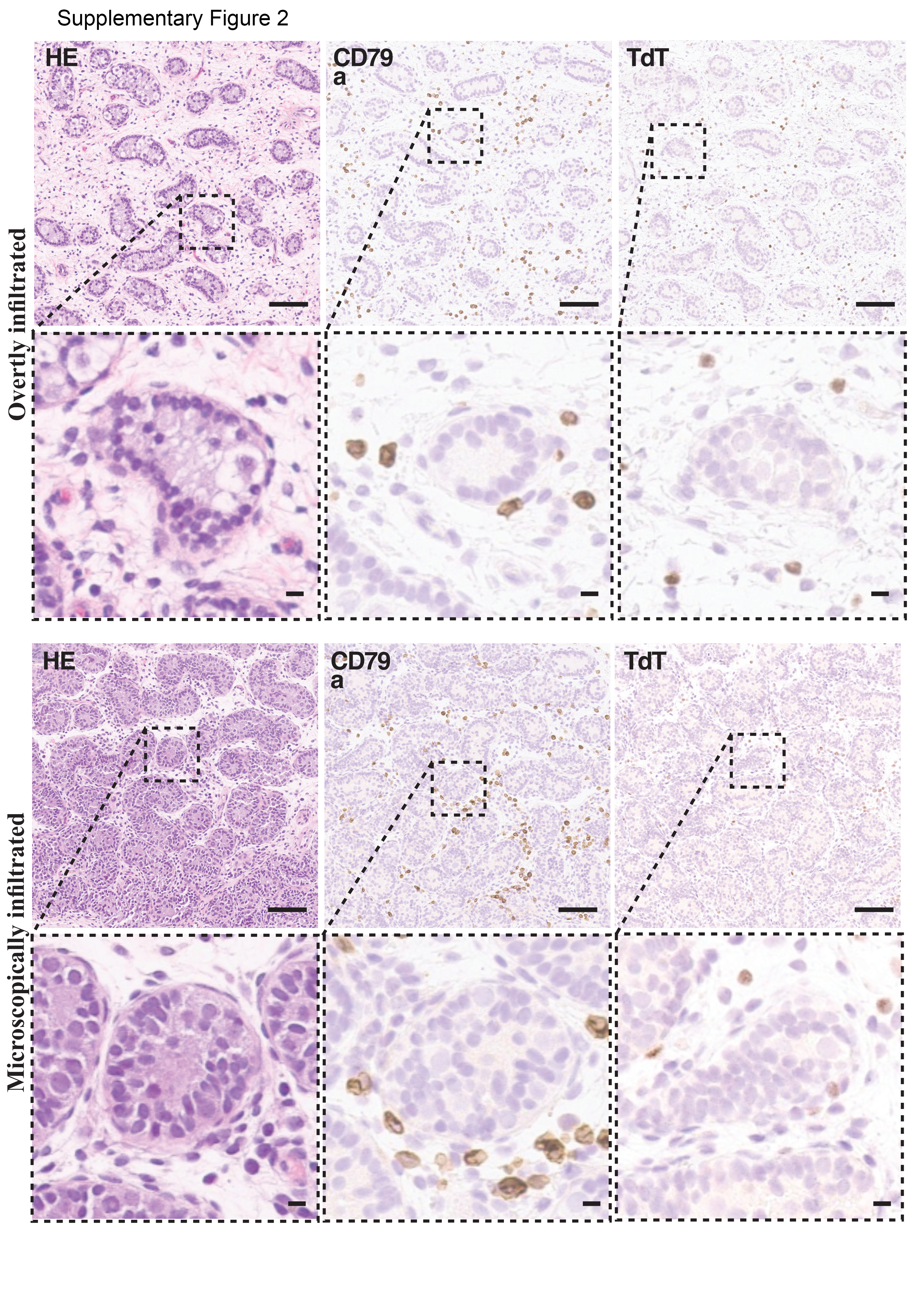

Supplement: Supplementary file 4 — Supplementary Figure S2 - Characterisation of human prepubertal testicular tissue microscopically and overtly infiltrated with leukaemic cells. [file 41375_2026_2938_MOESM4_ESM.tif]

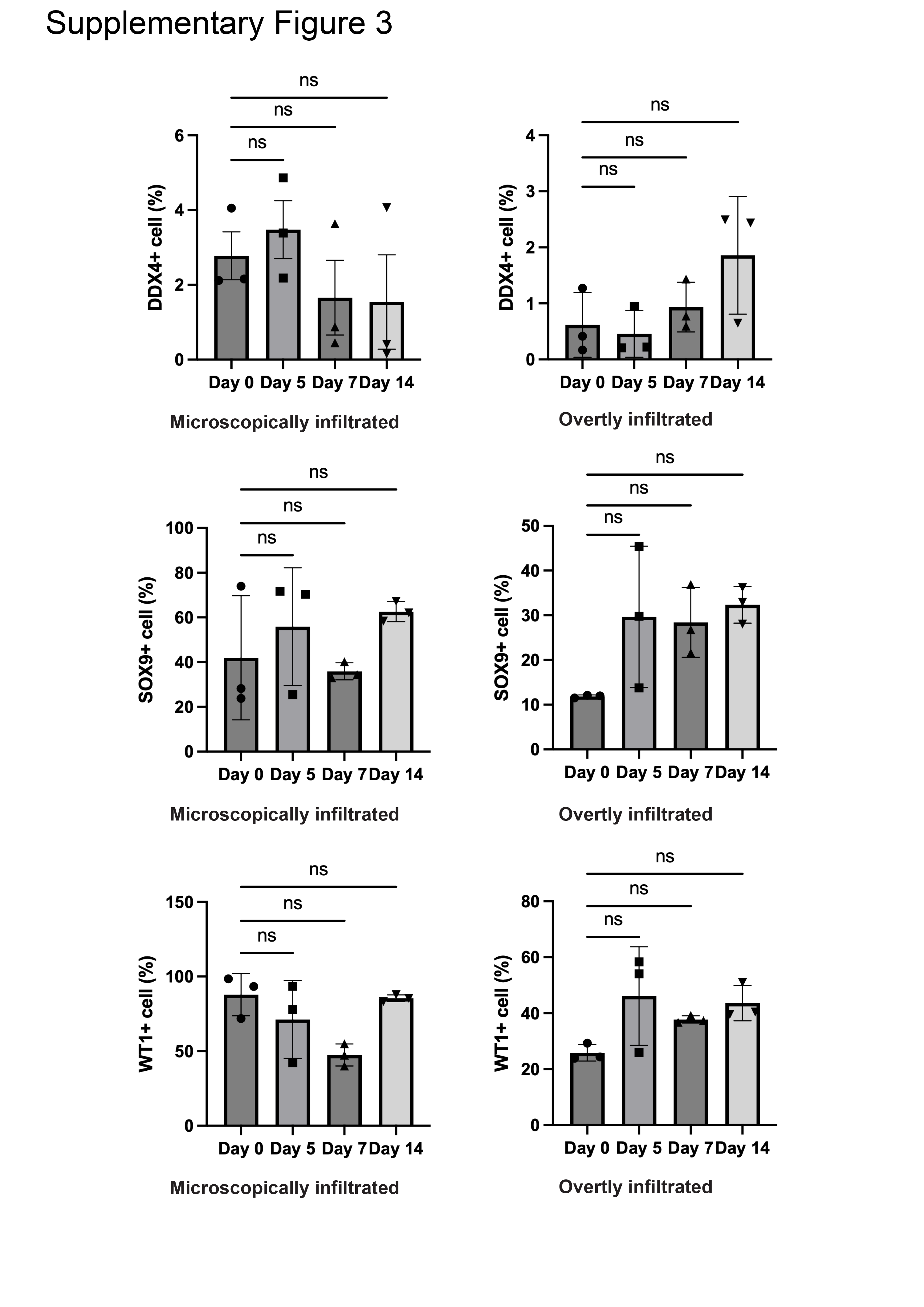

Supplement: Supplementary file 5 — Supplementary Figure S3 - Quantitative analysis of germ and Sertoli cell populations in organotypic cultures from microscopically or overtly infiltrated testicular tissue. [file 41375_2026_2938_MOESM5_ESM.tif]

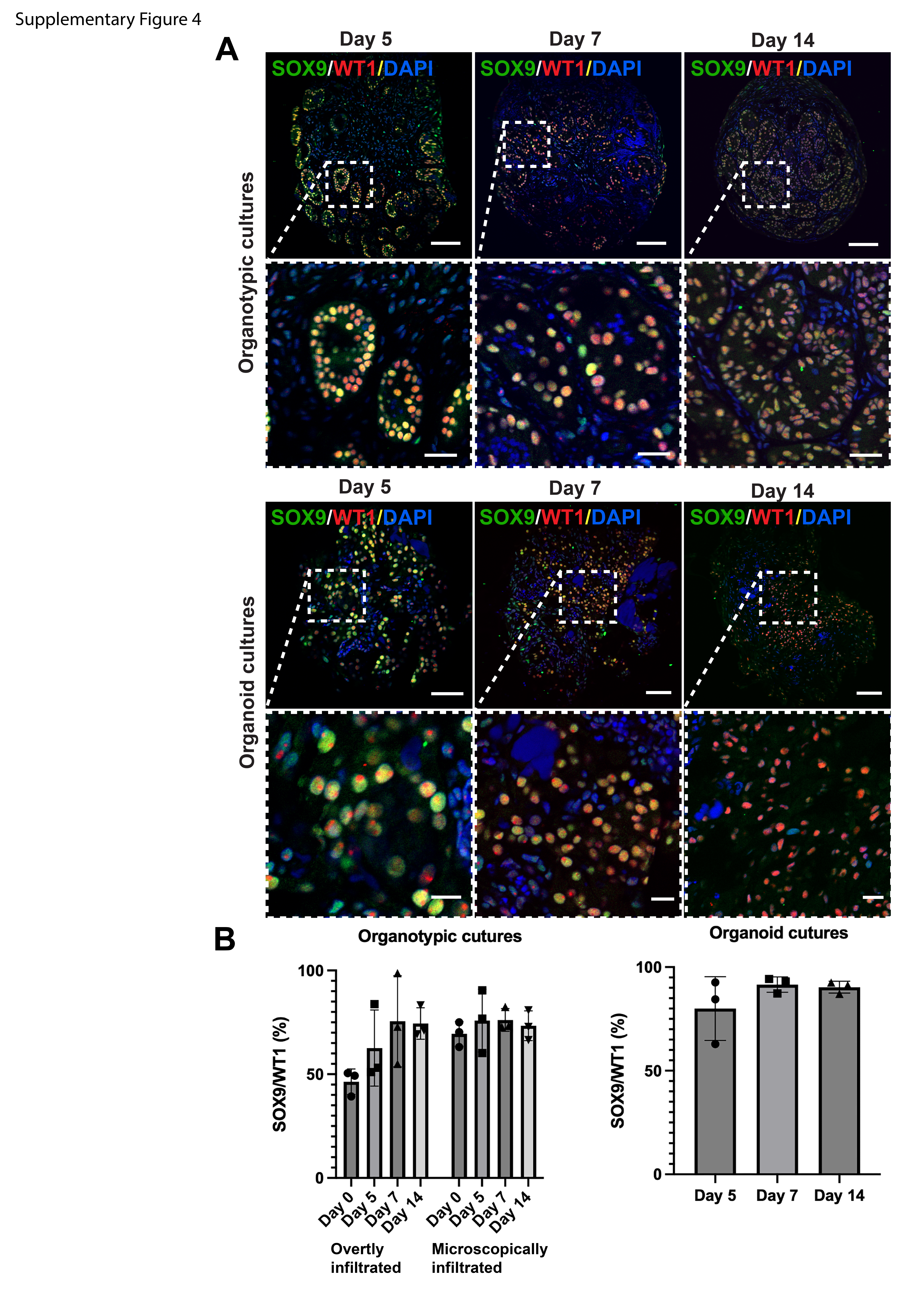

Supplement: Supplementary file 6 — Supplementary Figure S4 - Expression of WT1 and SOX9 in testicular organotypic and organoid cultures. [file 41375_2026_2938_MOESM6_ESM.tif]

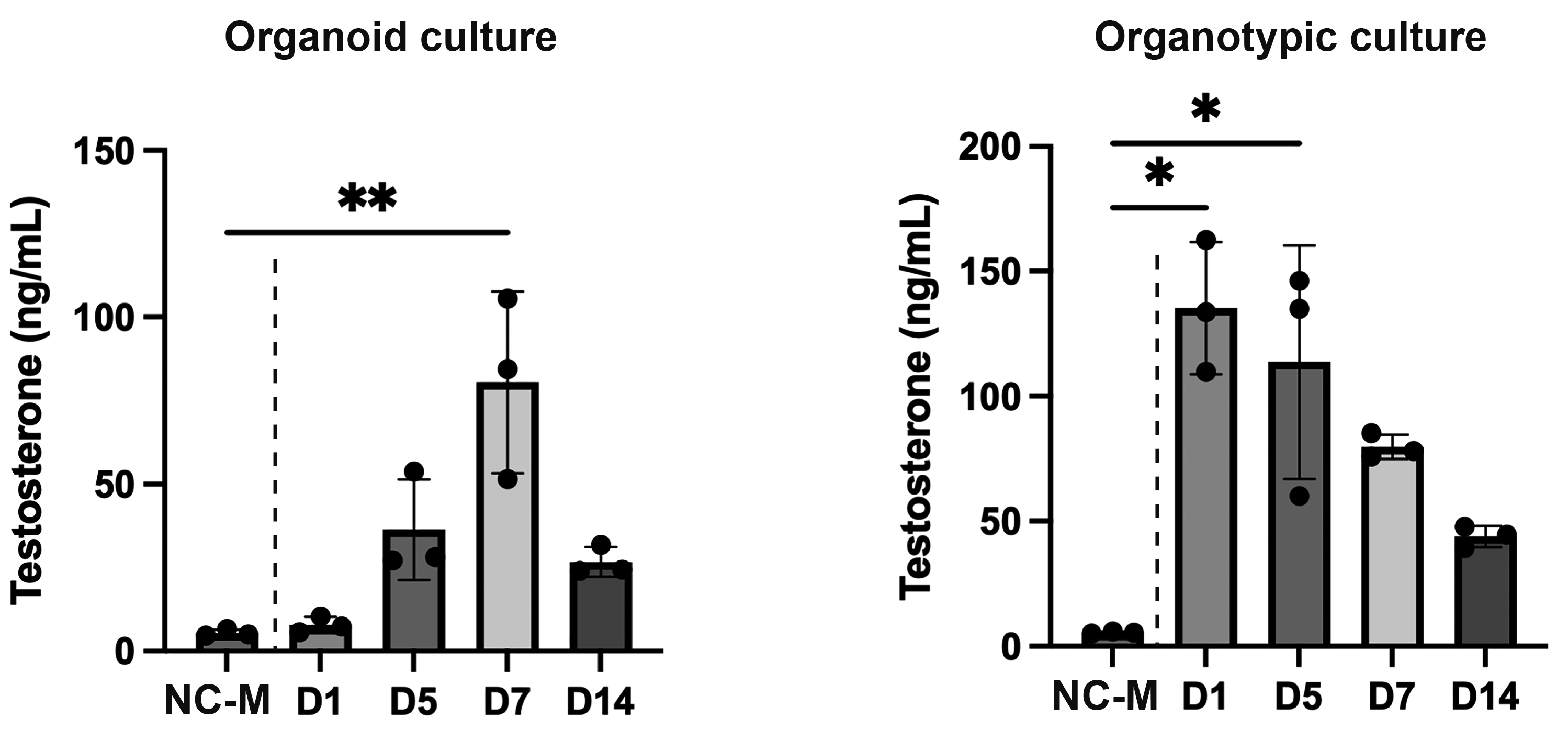

Supplement: Supplementary file 7 — Supplementary Figure S5 - Testosterone levels in organotypic and organoid cultures. [file 41375_2026_2938_MOESM7_ESM.tif]

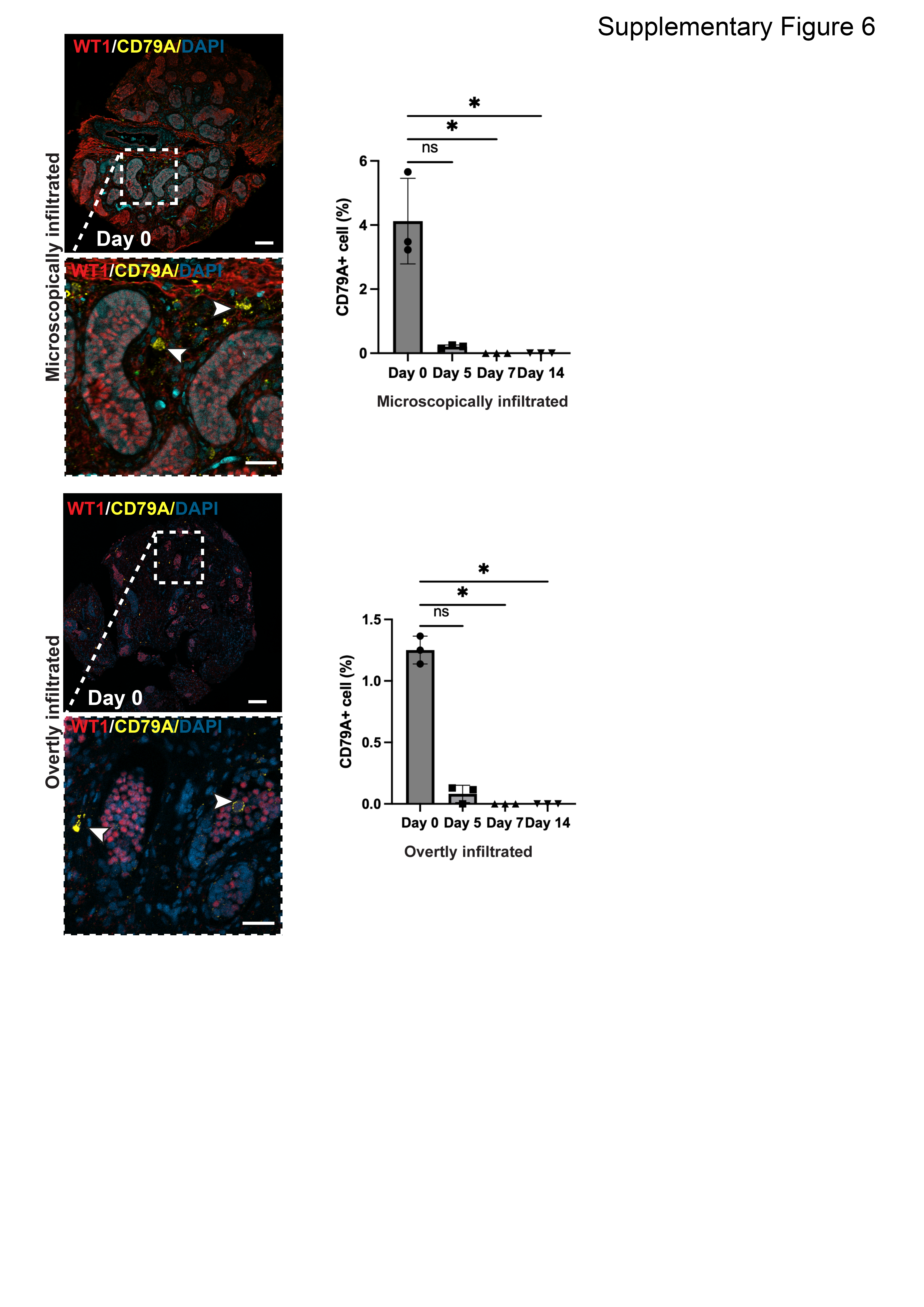

Supplement: Supplementary file 8 — Supplementary Figure S6 - Immunofluorescence analysis of leukaemic cell persistence in organotypic cultures. [file 41375_2026_2938_MOESM8_ESM.tif]
